# The Role of Chemokines and Chemokine Receptors in Diabetic Nephropathy

**DOI:** 10.3390/ijms21093172

**Published:** 2020-04-30

**Authors:** Ting-Ting Chang, Jaw-Wen Chen

**Affiliations:** 1 Department and Institute of Pharmacology, School of Medicine, National Yang-Ming University, Taipei 11221, Taiwan; tf0619@ym.edu.tw; 2 Department of Medical Research, Taipei Veterans General Hospital, Taipei 11217, Taiwan; 3 Cardiovascular Research Center, National Yang-Ming University, Taipei 11221, Taiwan; 4 Department of Medicine, Taipei Veterans General Hospital, Taipei 11217, Taiwan

**Keywords:** chemokine, chemokine receptor, diabetic nephropathy, diabetes mellitus, inflammation

## Abstract

Kidney function decline is one of the complications of diabetes mellitus and may be indicated as diabetic nephropathy (DN). DN is a chronic inflammatory disease featuring proteinuria and a decreasing glomerular filtration rate. Despite several therapeutic options being currently available, DN is still the major cause of end-stage renal disease. Accordingly, widespread innovation is needed to improve outcomes in patients with DN. Chemokines and their receptors are critically involved in the inflammatory progression in the development of DN. Although recent studies have shown multiple pathways related to the chemokine system, the specific and direct effects of chemokines and their receptors remain unclear. In this review, we provide an overview of the potential role and mechanism of chemokine systems in DN proposed in recent years. Chemokine system-related mechanisms may provide potential therapeutic targets in DN.

## 1. Background

Kidney failure is one of the major serious complications from diabetes mellitus (DM). About 30% to 40% of patients with diabetes develop diabetic nephropathy (DN), and DM accounts for 30% to 50% of the incident cases of end-stage renal disease (ESRD) in the United States [1]. DN develops in approximately 40% of diabetic patients. DN is a chronic disease that is typically characterized in clinical presentation by progressive proteinuria, hypertension, and a decreasing glomerular filtration rate (GFR) and results in the development of ESRD [2]. DN leads to a higher incidence of microangiopathy, cardiovascular events, and mortality. Hyperglycemia, acute kidney disease, hypertension, and obesity are risk factors of DN. Among these risk factors, hyperglycemia and hypertension are the most notorious [3]. Hyperglycemia and hyperfiltration result in morphological changes, such as mesangial expansion, extracellular matrix (ECM) accumulation, and glomerulosclerosis with nodular mesangial lesion [4]. Podocyte injury is an early event in DN. The number of podocytes is reduced in DN patients, and podocyte loss can act as a marker of progressive decline in renal function and proteinuria in DM [5,6]. Due to the global burden represented by DN, it is important to provide the best clinical treatment to patients with DN. 

Treatment strategies currently target glycemia, blood pressure, the renin–angiotensin system (RAS), blood cholesterol and so on, which can be initiated once abnormal albuminuria and renal function are detected [7]. The inhibition of the RAS with a single-agent angiotensin-converting enzyme inhibitor (ACEi), angiotensin receptor blockers (ARBs), or direct renin inhibitors reduces albuminuria, but does not significantly halt the progression of disease in patients with DN [8,9]. In addition, the prevention of excessive afferent arteriole dilation and reduction of the single nephron glomerular filtration rate to decrease hyperfiltration can only partially halt the disease progression [10]. While the contemporary management of diabetes, hypertension, and dyslipidemia could have limited impacts on the progression of DN, novel mechanisms should be discovered for advanced treatment in clinical settings.

Inflammation has been suggested to be a novel mechanism linked to the development of DN [11]. Macrophage infiltration in various tissues is a characteristic feature of both type 2 DM and DN. Macrophage accumulation plays a critical role in the progression of DN via the production of reactive oxygen species (ROS), cytokines, and proteases. Furthermore, kidney fibrosis results from ECM deposition, which is caused by the infiltration of immune cells, inflammatory cells, altered chemokines, and cytokine in the kidney [11]. Given the complexity of inflammation, it is therefore important to identify particular inflammatory pathways as potential therapeutic targets of DN. There are two major systems responsible for inflammation: cytokine and chemokine systems. While increasing numbers of studies have focused on the pro-inflammatory cytokines in the recent five years, the mechanistic role of chemokines in DN is not well known. Thus, this review mainly focuses on the role of chemokines in DN.

## 2. Chemokines and Chemokine Receptors

Chemokines, small molecular weight chemotactic cytokines, and their receptors play key roles in the recruiting and physiological direction of cell migration. They can be broadly divided into four subfamilies—the CC, C, CXC, and CX3C families—according to their N-terminal cysteine-motifs [12]. The CXC subfamily is further divided into two parts: one contains an ELR (glutamic acid–leucine–arginine) motif preceding the first cysteine, and the other does not [13]. Chemokines are also defined as “homeostatic” chemokines and “inflammatory” chemokines according to their functions. Homeostatic chemokines are constitutively secreted and mainly involved in lymphocyte traffic; inflammatory chemokines are related to pro-inflammatory mechanisms and induce leukocyte recruitment to damaged tissues [14].

## 3. The Role of Chemokines in Diabetic Nephropathy

Chemokines are shown to be closely related to DN in clinical settings. Mesenchymal stem cells can orchestrate local and systemic innate and adaptive immune responses through the release of chemokines when exposed to an inflammatory environment in DN [15]. In DN animal models, numerous chemokines in different families, such as CCL2, CCL20, CXCL5, CXCL7, and CXCL12, are increased in glomeruli and proximal tubules [16,17]. A number of chemokines are discussed in detail in the following paragraphs.

### 3.1. The Potential Role of CCL2 in Diabetic Nephropathy

The most well-known CC chemotactic chemokine family is CCL2, also called macrophage chemokine-1 (MCP-1), which is suggested to reflect the level of tubular injury and renal inflammation in DN [18,19,20]. The established risk biomarkers include CCL2, matrix metallopeptidases (MMPs), tyrosine kinase, podocin, connective tissue growth factor, tumor necrosis factor (TNF)-receptor-1, sclerostin, YKL-40, and NT-proCNP, which improve the explanations and prediction of eGFR decline in type 2 diabetes [21]. CCL2 modulates the recruitment of inflammatory cells and the differentiation of macrophages. In macrophages, high glucose activates macrophages via transforming growth factor-β (TGF-β)-activated kinase 1 (TAK1)/mitogen-activated protein kinase (MAPK) and TAK1/nuclear factor (NF)-κB-dependent pathways, which lead to the polarization of macrophages to a pro-inflammatory phenotype and accelerate DN [22].

Several studies indicate that DN could be attenuated or improved in combination with decreased levels of CCL2, suggesting the potential role of CCL2 in the progression of DN [16,17,23,24]. High glucose induces the up-regulation of toll-like receptors (TLR) 4 and CCL2 through nuclear factor-kappa B (NF-κB)-dependent signaling in podocytes [25]. The activation of TLRs stimulates the expression of inflammatory cytokines and chemokines including CCL2 and TNF-α, and this is closely linked with the progression of DN [26]. Another study has also revealed that high glucose enhances CCL2 expressions and activates the phosphorylation of NF-κB/p65, IκB kinase (IKK) β, IκBα, MAPK, and the nuclear translocation of p65 in podocytes. The expressions of receptor activator for NF-κB (RANK) are up-regulated in high glucose-induced podocytes, and RANK siRNA attenuates high glucose-induced podocyte injury by inhibiting the activation of NF-κB and MAPK signaling pathways [27]. Moreover, advanced glycation end products (AGEs) or dipeptidyl peptidase-4 (DPP-4) can induce the up-regulation of NF-κB p65 or CCL2 mRNA levels in tubular cells [28]. Aldosterone can induce an inflammatory response through the activation of NF-κB and CCL2 in mesangial cells under the high glucose condition via the angiotensin II receptors pathways [29]. High glucose can induce the phosphorylation and sumoylation of IKK*γ* and activated NF-*κ*B signaling, accompanied by up-regulated CCL2 and IL-6 [30]. Furthermore, angiotensin II synergizes with high glucose in the release of pro-inflammatory factors, such as CCL2 and IL-6 via the activation of TLR4 signaling [31]. In human kidney-2 (HK-2) cells, high glucose treatment induces interleukin (IL)-6 and CCL2 in a dose and time-dependent manner [32]. Fetuin-A or lipopolysaccharide (LPS) exacerbates palmitic acid-induced podocyte death, accompanied by the up-regulation of CCL2 and keratinocyte chemoattractant [33]. The lack of semaphorin 3G, a glomerulus-specific transcript belonging to the semaphorin family, results in the enhanced expression of CCL2 and IL-6 with impaired foot process structures in podocytes under DN conditions [34]. 

High glucose or TGF-β1-induced IL-20 leads to apoptosis by activating caspase-8. Meanwhile, IL-20 can up-regulate MMP-9, CCL2, TGF-β1 and vascular endothelial growth factor (VEGF) expression in podocytes [35]. TGF-β1 also increases CCL2 and MCP-1 induced protein-1 (MCPIP1), a suppressor of microRNA (miR)-146a via the ErbB4/epidermal growth factor receptor signaling pathway [36]. In glomerular mesangial cells, the up-regulated expression of endothelial vascular cell adhesion protein 1 (VCAM-1) and CCL2 can be sustained for at least 72 h under high glucose conditions [37]. Advanced glycation end products (AGEs) increase the expression of intercellular adhesion molecule 1 (ICAM-1) and CCL2 through the member A Rho kinase (RhoA/ROCK) signaling pathway [38]. In addition, P2X7 receptors are expressed on macrophages and are major components of pro-inflammatory signaling. P2X7 receptor activation leads to the release of CCL2 under high glucose conditions [39]. Accelerated ALPK1 expression up-regulates renal CCL2 and CCL5 expressions in streptozotocin (STZ)-induced DN mice in vivo and in HK-2 cells in vitro [40]. 

### 3.2. Modulation of CCL2 in Experimental Diabetic Nephropathy

The inhibition of TLR4 prevents the release of CCL2 and keratinocyte chemoattractant and decreases the podocyte death induced by palmitic acid alone or in combination treatment with Fetuin-A [33]. Moreover, TAK1 inhibition not only decreases high glucose-induced CCL2 and TNF-α, but suppresses ERK1/2, p38 MAPK phosphorylation and nuclear factor-kappa B (NF-κB) activation [41]. In glomerular endothelial cells, a neutralizing anti-CCL2 antibody can prevent VCAM-1 up-regulation [42]. In renal tubular epithelial cells, metformin can prevent TGF-β1-induced CCL2 expression through the regulation of bone morphogenetic protein and activin membrane-bound inhibitor (BAMBI)-mediated inhibition of mitogen-activated and extracellular signal-regulated kinase kinases 1/2 (MEK-1/2) and the extracellular signal-regulated kinases 1/2 (ERK1/2) signaling pathway [43]. CCL2 gene expression and cell apoptotic levels are enhanced under high glucose conditions, which could be attenuated by the sodium–glucose cotransporter 2 (SGLT2) inhibitor tofogliflozin or antioxidant N-acetylcysteine treatments in human proximal tubular cells [44]. 

In STZ-induced DN mice, a glucagon-like peptide-1 (GLP-1) analog attenuates the levels of ROS, proinflammatory cytokine and chemokine including TNF-α, IL-1β, CCL2, ICAM-1, and fibrosis-related molecules including TGF-β1 and fibronectin with reduced tubular injury and macrophage infiltration [45]. Furthermore, enhanced renal fibrosis, mesangial proliferation, podocyte loss, TGF-β, CCL2 expressions, and suppressed Rho levels are observed in renal tissues in STZ-induced DN rats. The treatment of Pitavastatin, an HMG-CoA reductase inhibitor, can ameliorate the above indices and exhibit reno- and podocyte-protective effects [46]. On the other hand, the inhibition of high mobility group box 1 (HMGB1) reduces CCL2, ICAM-1, TGF-β1, receptor for advanced glycation end products (RAGE) and TLR4 expressions in the kidney tissue [47]. The SGLT-2 inhibitor can down-regulate NF-κB activity and reduce the expression of TNF-α and CCL2 in renal cortices as well as the levels of IL-6 and alpha-1 acid glycoprotein (AGP) in urine [48]. H2AK119 monoubiquitination regulates both Type 1 and Type 2 receptors of angiotensin II-mediated macrophage infiltration through CCL2 in type 2 diabetic rats [49]. In high-fat diet and STZ-induced DN rats, berberine and Tangshen Formula can not only inhibit the up-regulation of IL-1β, TNF-α, and CCL2 by inactivating the NF-κB signaling pathway, but also attenuate renal fibrosis via the TGF-β/Smad3-mediated signaling pathway [50,51,52]. In alloxan-induced diabetic rabbits, lycium barbarum polysaccharides can decrease DM-induced levels of CCL2 and ICAM-1 mRNA by down-regulating the expression of NF-κB and angiotensin II in the kidneys and protecting renal function in DN [53]. In db/db mice, TAK1 inhibitor reduces the DM-induced macrophage infiltration and inflammatory protein expressions in renal tissues [41]. In db/db mice, the *Dianthus superbus*-EtOAc soluble fraction has a renoprotective effect with decreasing albumin excretion, plasma creatinine, kidney injury molecules-1 (KIM-1), C-reactive protein, TGF-β, and CCL2 levels. It can also directly reduce inflammation and fibrosis in human renal mesangial cells [54]. More interestingly, in diabetic Apolipoprotein E knockout mice, the inhibition of CCL2 reduces albuminuria, restores glomerular endothelial glycocalyx and barrier function, and reduces tissue inflammation [55].

The NADPH oxidase-derived production of ROS in the kidney plays a role in modulating DN. The vascular smooth muscle cell and mesangial cell-specific overexpression of Nox5 isoform of NADPH oxidase leads to increased glomerular ROS production, glomerulosclerosis, mesangial expansion, and ECM accumulation with increased macrophage infiltration and CCL2 levels in a mouse model of DN [56]. Additionally, oral iron chelator deferiprone has a renoprotective effect by decreasing ROS levels and reducing inflammation and fibrosis markers, such as NF-κB, CCL2, MMP-9, tissue-specific inhibitor of metalloproteinase (TIMP)-1, cyclo-oxygenase (COX)-2, and nitrotyrosine in kidney tissues in high-carbohydrate-fat diet and STZ-induced DN rats [57]. A screened DNA aptamer directed against RAGE suppresses the AGE and RAGE-induced ROS and CCL2 levels in the kidneys and improves podocyte injury and albuminuria in diabetic rats [58].

### 3.3. Modulation of CCL2 in Clinical Diabetic Nephropathy

In a clinical setting, the CCL2-2518 GG genotype and G allele increase the risk of progression to ESRD in type 2 DM [59]. Elevated urinary CCL2 levels are observed before clinical findings of DN in women with type 1 DM. The CCL2 concentration changes are associated with the kidney interstitial volume, suggesting that inflammatory processes are related to the pathogenesis of early interstitial changes in DN [60]. Serum levels of CCL2 and plasminogen activator inhibitor type 1 levels are associated with inflammatory and fibrotic processes in the kidneys in DN patients [61]. CCL2 was up-regulated in kidney tissue samples from DN patients. Furthermore, miR-374a can negatively regulate CCL2 expression in human renal tubular epithelial cells [62]. Urinary values of CCL2 are shown to be an important biomarker in predicting rapid decline in DN patients [63,64]. Additionally, urine levels of the CCL2-to-creatinine ratio are shown to be associated with sustained renal decline in type 2 DM patients with preserved renal function [65]. Urinary CCL2 levels are increased and are positively correlated with the number of macrophages in the renal interstitium and with the levels of tubulointerstitial lesions, suggesting that CCL2 expressions may be locally related to the development of DN [66,67,68]. 

In clinical trials, the CCL2 inhibitor emapticap pegol and the Janus kinase (JAK)1/JAK2 inhibitor baricitinib seem to be promising drugs for DN [69]. Baricitinib down-regulates urine CCL2 expression with a decreased urine albumin–creatinine ratio (UACR) in type 2 diabetes patients at high risk for progressive DN in a phase 2 randomized controlled clinical trial [70]. Furthermore, phase 2 clinical trials evaluating the inhibitors of CCL2 have produced promising results for UACR and HbA1c, and these might be promising therapeutic targets for DN [71,72]. 

### 3.4. Summary of CCL2 in Diabetic Nephropathy

In summary, CCL2 has direct signaling effects on cell migration, proliferation, and differentiation [19]. The inhibition of CCL2 can ameliorate the disease in different in vitro and in vivo models as well as in clinical trials of renal disease, suggesting that the inhibition of CCL2 may be a promising strategy to treat patients with renal inflammatory disease. The beneficial effects of CCL2 blockade may mainly arise from inactivating the NF-κB signaling pathway.

### 3.5. Other Members of CC Chemotactic Chemokine Family in Diabetic Nephropathy

Previous studies also provided the explanation for the role of other chemokines in the CC chemotactic family in DN. For example, plasma levels of plasminogen activator inhibitor-1, syndecan-1, VEGF, IL-1β, interleukin-1 receptor antagonist, and CCL4 are increased in type 1 DM patients with kidney failure [73]. The renal expression of CCL5, also known as RANTES, is increased with fibrosis and inflammation during kidney injury in STZ-induced DM mice [74]. CCL7 was previously called monocyte-chemotactic protein-3 (MCP-3), which could be up-regulated in early renal injury in adolescent patients with type 1 diabetes [75]. The combination treatment of high glucose and recombinant CCL18 increases fibronectin expressions compared to high glucose treatment alone in HK-2 cells [76]. Linagliptin, a DPP-4 inhibitor, reduces CCL2, CCL22, and IL-12 in type 2 diabetes patients with or without DN compared with the placebo group, suggesting its anti-inflammatory abilities in type 2 DM [19]. Taken together, the above chemokines in the CC chemotactic family are positively related to DN. However, the determination of whether they have a direct role in the pathology of DN or whether they can modulate the disease still needs further research.

### 3.6. Members of Chemokine CXC Family in Diabetic Nephropathy

Several members of the chemokine CXC family, including CXCL1, 7, 8, 9, 10, 11, 12, and 16, may be involved in diabetic nephropathy. The expression of CXCL1 is increased in podocytes of OVE, db/db and STZ diabetic mouse models [77]. 

The glomerular endothelium dysfunction plays an important role in the pathogenesis of early DN. In primary rat glomerular endothelial cells, platelet microparticle-derived CXCL7 results in glomerular endothelial injury, which can be reversed by CXCL7 inhibition with a CXCL7 neutralizing antibody [78].

CXCL8 inhibition improves the urine volume, urine albumin/creatinine ratio, blood urea nitrogen, and creatinine clearance rate with decreased mesangial expansion, glomerulosclerosis, and extracellular matrix deposition in DN mice. Furthermore, CXCL8 inhibition attenuates high glucose-induced inflammatory and fibrotic-related proteins in human renal mesangial cells [79]. 

Serum and urine levels of CXCL9 are enhanced in patients with DN compared with healthy controls. Urinary CXCL9 and CXCL11 mRNA levels correlate with baseline renal function. The rate of renal function decline is associated with urinary CXCL9 mRNA level in DN [80]. AGEs decrease the proliferation of mouse podocytes and increase the expression of CXCL9 through the activation of signal transducer and activator of transcription 3 (STAT3). The knockdown of CXCL9 leads to the decreased expressions of Bax/Bcl-2 and inactivation of the Janus kinase 2 (JAK2)/STAT3 pathway, resulting in anti-apoptotic and anti-inflammatory effects in a model of mouse podocyte injury of DM [81]. 

Plasma CXCL10 levels are decreased in diabetic db/db mice compared with non-diabetic mice; nevertheless, the concentrations of CXCL9 and CXCL11 are similar. Furthermore, recombinant murine CXCL10 can reduce mesangial and peritubular matrix expansion, albuminuria, and glomerular hypertrophy in vivo. In the in vitro part, CXCL10 can inhibit kidney fibroblast collagen and TGF-β production under high glucose conditions [82]. In contrast, a proteoglycan of the extracellular matrix stimulates the production of Th1 and Th17 chemoattractants CXCL10 and CCL20 in macrophages. Biglycan triggers the expression of CXCL9 and CXCL10, chemoattractants for CXCR3-positive Th1 and Th17 cells, via the TLR4/TRIF-dependent pathway. Biglycan also induces CCL20 chemokine production, chemoattractants for CCR6-positive Th17 cells, via the TLR2/4/MyD88-dependent pathway. In DN patients, a positive correlation of CXCL9 and CXCL10 levels is detected in plasma [83]. Baricitinib, an oral JAK1 and JAK2 inhibitor, was shown to down-regulate the urine CXCL10 level with decreased UACR in a phase 2 clinical trial in DN patients [70]. 

DPP-4 inhibitor increases GLP-1 and CXCL12 in type 2 diabetes patients with or without DN compared with the placebo group, suggesting its pro-vasculoregenerative ability in type 2 DM [19]. The DPP-4 inhibitor, but not the glucagon-like peptide-1 receptor (GLP-1R) agonist, enhances the renal CXCL12 expression in GLP-1R knockout diabetic KK/Ta-Akita mice. In wild-type diabetic mice, DPP-4 inhibitor treatment can reverse the increased levels of renal CXCL12, albuminuria, glomerulosclerosis, fibrosis, podocyte loss, and ROS. Taken together, DPP-4 inhibition may provide renal protection effects via CXCL12-dependent and GLP-1R-independent signaling pathways in DN [84]. However, the direct activation of proximal tubular cells by uric acid is shown to result in enhanced release of CXCL12 and HMGB1 and accelerated macrophage recruitment [85]. 

Compared with untreated db/db mice, db/db mice, which received casein, have accelerated tubulointerstitial injury with increased secretion of ECM and cholesterol accumulation in tubulointerstitium, which is accompanied by the induction of the CXCL16. In HK-2 cells under high glucose conditions, CXCL16 knocked down by siRNA can reverse IL-1β-induced cholesterol accumulation, ROS production, and ECM protein expression [86]. Increased plasma CXCL16 levels can be related to decreased eGFR and elevated albuminuria values in European patients with DN [87]. In patients with type 2 diabetes enrolled in the ROADMAP (Randomized Olmesartan and Diabetes Micro-Albuminuria Prevention) and observational follow-up studies, enhanced plasma CXCL16 levels, angiopoietin-2, and TGF-β1 were shown to be independently predictive of microalbuminuria [88]. Urinary CXCL16 and endostatin can reflect the degree of interstitial fibrosis and tubular atrophy, which is a risk factor for poor renal outcome in DN patients, and act as biomarkers among these patients [89]. 

In summary, anti-chemokines may be potential therapeutic targets due to the majority of chemokines in the CXC family seeming to be pro-inflammation and related to disease progression in DN. Nevertheless, the roles of CXCL10 and CXCL12 are multifaceted.

### 3.7. Members of Chemokine CX3C Family in Diabetic Nephropathy

In the chemokine CX3C family, CX3CL1, also known as fractalkine, is mainly produced by glomerular endothelial cells and the tubular epithelium, which can be also detected in podocytes, mesangial cells, renal tumor cells and stromal cells [90]. CX3CL1 is expressed in inflammatory renal tissues in humans [91,92]. CX3CL1 can induce mesangial ECM via the activation of its receptor CX3CR1, ROS and MAPKs. A TGF-β antibody can attenuate the expression of CX3CL1 under high glucose conditions and decrease CX3CL1-induced ECM accumulation in mouse mesangial cells [93]. Based on the above observations, CX3CL1 is linked with the pathological changes in DN, and future investigations in the chemokine CX3C family may be worthwhile, although only few research works have focused on this family.

## 4. The Role of Chemokine Receptors in Diabetic Nephropathy

### 4.1. The Potential Role of CCR2 in Diabetic Nephropathy

Inflammatory cell recruitment, infiltration, and activation play a critical role in the development of DN. CCL2 can activate C-C chemokine receptor type 2 (CCR2) to stimulate the release of monocytes from the bone marrow and lead to the recruitment of monocytes to inflammation area [94]. The major receptor for CCL2 on monocytes is CCR2 [95]. The CCL2/CCR2 system is a key pro-inflammatory system in DN [96]. The accumulation of CCR2-expressing macrophages promotes renal injury and fibrosis in DN [97]. 

In an STZ-induced diabetic mouse model with hypertension, the combination treatment of CCR2 antagonist and ACEi exhibits better renal protection than ACEi alone [98]. In diabetic db/db mice, CCR2 inhibition by a small-molecule antagonist can attenuate proteinuria, glomerulosclerosis, and kidney failure [99]. In CCR2 knockout mice with DN, glomerular VCAM-1 expressions are decreased. CCL2/CCR2 signaling can induce glomerular VCAM-1 up-regulation via the p38 MAPK, mitogen- and stress-activated protein kinases 1/2 (MSK1/2), and phosphorylation of H3Ser10 pathways [42]. In the CCR2 knockout and STZ-induced diabetic nephropathy-prone (DBA/2J) mouse model, kidney protection effects are observed after nine weeks of diabetes. In addition, diabetic mice expressing CCR2 specifically in podocytes of CCR2 knockout mice have increased albuminuria, blood urea nitrogen (BUN), fibronectin and type 1 collagen expression, podocyte loss, and glomerular apoptosis [100].

In DN patients, CCR2 expression is up-regulated by glomerular podocytes, suggesting the potential role of CCR2 beyond the recruitment of monocytes [101]. A previous clinical study also shows the beneficial effects of CCR2 inhibitor CCX140-B on DN [69]. Inhibitors of CCL2/CCR2 pathways may be promising therapeutic options to improve renal outcomes in DN patients, but more clinical trials are necessary [102]. Taken together, targeting the CCR2 signaling pathway in podocytes may be a potential therapeutic approach for the treatment of DN.

### 4.2. The Potential Role of CCR5 in Diabetic Nephropathy

C-C chemokine receptor type 5 (CCR5) is a receptor for CCL5. Increased CCR5 expressions are observed in inflammatory kidney diseases and transplant rejection [103]. However, the role of CCR5 in DN is nearly unexplored. A previous study indicates that the presence of a CCR5 polymorphism is related with better outcomes in type 2 diabetes patients, suggesting that the blockade of CCR5 may be a potential treatment strategy for DN [104]. In a phase 2 study, a dual CCR2 and CCR5 receptor antagonist was developed for the treatment of DN. After dual CCR2 and CCR5 receptor antagonist treatments, the urinary albumin-to-creatinine ratio was reduced in type 2 diabetes patients who also received standard of care, such as ACEi or ARB [105]. However, dual CCR2 and CCR5 receptor antagonist treatments exhibited no beneficial effects on kidney disease progression in ob/ob mice. It seems that the CCR signaling blockade is ineffective under severe hyperglycemia conditions [106]. In addition, evidence of the mono-blockade of CCR5 is lacking.

### 4.3. Chemokine CXC Motif Receptors in Diabetic Nephropathy

Chemokine CXC motif receptor (CXCR) 1 and CXCR2 inhibitors can reverse CXCL7-induced injury in primary rat glomerular endothelial cells [78]. CXCL8 binds to CXCR1 and CXCR2 to recruit neutrophil infiltration and induce tissue inflammation. CXCL8 inhibition blocks the activation of CXCR1 and CXCR2 and their downstream JAK2/STAT3 and ERK1/2 pathways in in vitro and in vivo DN models [79]. CXCR3 is the cognate receptor for CXCL10. Genetic variants in apolipoprotein L1 (APOL1) gene are linked with the up-regulation of RNA encoding CXCR3 ligands and affect the outcomes of DN [107]. CXCR3 silencing leads to abolished anti-fibrotic effects from CXCL10, indicating that CXCL10 abundance is necessary to prevent fibrosis and the progression of DN in experimental diabetes [82]. On the other hand, AGEs can up-regulate the expression of both CXCL9 and CXCR3 via the STAT3 signaling pathway in podocytes [81]. The binding of the receptor CXCR4 to its ligand CXCL12 plays a role in the development and in pathological growth of renal cells. Renal CXCR4 expressions are increased in diabetic rats and in biopsy tissue from patients with DN; however, CXCR4 inhibition further enhances albuminuria and accelerates tubular epithelial cell death. CXCR4 inhibition promotes cell apoptosis, activates Bcl-2-associated cell apoptosis promoter, and attenuates the high glucose and CXCL12-augmented phosphorylation of pro-survival signaling in tubular epithelial cells [108]. According to recent studies, it seems that the blockade of some receptors in the CXCR family might be helpful for DN. However, more factors must be considered due to the multiple and complex effects of the receptors in this family.

### 4.4. Chemokine CX3C Motif Receptors in Diabetic Nephropathy

Chemokine (CX3C motif) receptor 1 (CX3CR1) is expressed on mononuclear cells, lymphatic leucocytes and especially in inflammatory renal tissues [109,110]. A high number of CX3CR1-positive inflammatory cells was found in different diseases, suggesting that it has a role in the progression of diseases [111]. The CX3CL1 and CX3CR1 axis is activated by several cytokines, including interferon-γ (INF-γ), IL-1, IL-6, IL-10, and TNF-α [112,113]. In STZ-induced diabetic mice, CX3CR1 knockout mice show lower levels of renal inflammation, fibrosis and ECM including in the decreased fractional mesangial area, fibronectin and collagen expressions compared with diabetic wild-type mice. High glucose and TGF-β1 can activate both CX3CL1 and CX3CR1 expressions accompanied with higher expression of ECM proteins in mouse mesangial cells, and high glucose and TGF-β1-induced effects are reversed by CX3CL1 or CX3CR1 siRNA [93]. It seems that the CX3CL1/CX3CR1 axis can be regarded as a novel perspective to the treatment of DN [111]. However, the DPP-4 inhibitor linagliptin increases the CX3CR1-positive monocytes with enhanced putative vasculoregenerative cells in type 2 diabetes patients with or without DN [19]. Therefore, whether the CX3CR1 can act as a novel target to the treatment of DN still needs further investigation.

Moreover, the atypical chemokine receptor ACKR2 is a chemokine scavenger receptor for several CC chemokines, which does not induce typical downstream G-protein mediated signaling pathways. ACKR2 can bind CC-chemokines, such as CCL2, CCL5, CCL3, CCL4, CCL7, CCL8, CCL11, CCL13, CCL17, CCL22, CCL23, and CCL24, and then leads to their degradation and reduces local inflammatory chemokine expressions as a result [114,115]. Levels of CCL2 and CCL5 mRNA are increased in OVE mice compared to OVE-ACKR2 knock-out mice. In OVE diabetic mice, ACKR2 deletion reduced albuminuria, renal inflammation, and renal fibrosis [116]. Further research is required to determine the clinical impacts of ACKR2 inhibition on DN.

## 5. Conclusions

**Figure 1:**
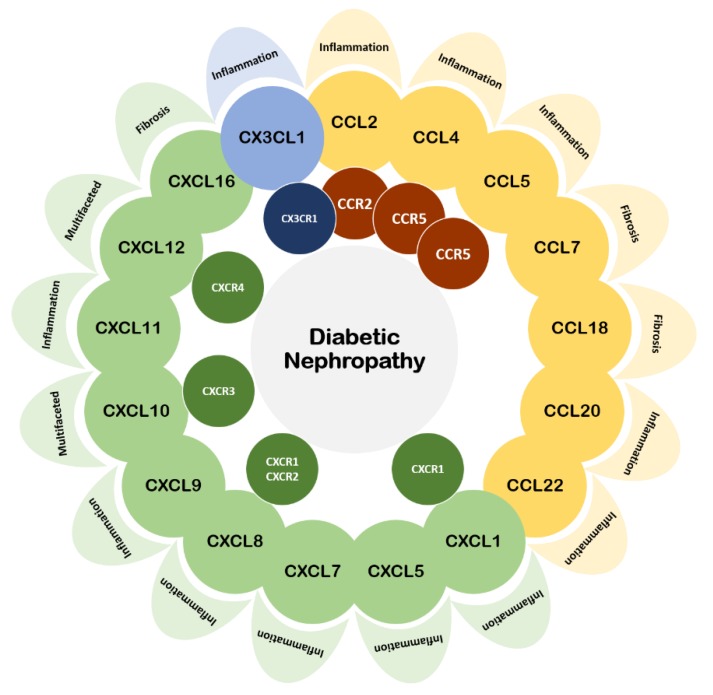
Correlation of the chemokines and their receptors in diabetic nephropathy discussed in this review article. Most of them are pro-inflammation or pro-fibrosis in diabetic nephropathy.

In this review article, we have cited most studies related to chemokines and chemokine receptors in DN over the past five years (Table 1). Recent findings demonstrated that chemokines and their receptors play diverse roles in the inflammatory events underlying DN (Figure 1). Most of them are positively related to the progression of the disease. Increased expressions of chemokines and their receptors are usually observed with declined DN outcomes. Chemokines seem not only to be the biomarkers but also potential therapeutic targets of DN. Pathophysiological changes, such as enhanced podocyte apoptosis, increased expressions of adhesion and fibrosis molecules, activated inflammatory responses, and infiltrated immune cells, are observed in DN. The inhibition of chemokines may retard the disease progression mostly via their anti-inflammatory or anti-fibrotic effects. In this context, the CCR2–CCL2 axis is the most well-known and well-investigated target for DN treatments. However, our understanding of the complex communication system among chemokines and their receptors is still deficient, which may hinder the development of novel therapeutic approaches in DN. If possible, the role of each chemokine and receptor should be fully and directly investigated to ensure translation to potential clinical implications. As a result, further experimental and clinical studies are required to clarify whether an anti-inflammatory strategy targeting particular chemokines and/or chemokine receptors could be a promising therapeutic approach to retard the progression of DN.

## Figures and Tables

**Table 1:** Summary of the chemokines and their receptors in diabetic nephropathy discussed in this review article. DM: diabetes mellitus; DN: diabetic nephropathy.

Studies of Chemokines and Their Receptors in Diabetic Nephropathy		
Subfamily	Chemokine/ Main Features	Chemokine Receptor
**CC**	CCL2 [16,17,18,19,20,21,22,23,24,25,26,27,28,29,30,31,32,33,34,35,36,37,38,39,40,41,42,43,44,45,46,47,48,49,50,51,52,53,54,55,56,57,58,59,60,61,62,63,64,65,66,67,68,69,70,71,72]/ reflect tubular injury and renal inflammation; modulate macrophage differentiation CCL4 [73]/ up-regulated in type 1 DM patients with kidney failure CCL5 [74]/ up-regulated in renal fibrosis and inflammation CCL7 [75]/ up-regulated in early renal injury CCL18 [76]/ increase fibronectin expressions CCL20 [16,83]/ up-regulated in glomeruli and proximal tubules CCL22 [19]/ down-regulated after DPP-4 inhibition	CCR2 [42,69,94,95,96,97,98,99,100,101,102,105,106]/ promote renal injury and fibrosis CCR5 [103,104,105,106]/ correlated with renal inflammation
**CXC**	CXCL1 [77]/ up-regulated in podocytes CXCL5 [16]/ up-regulated in glomeruli and proximal tubules CXCL7 [16,78]/ participate in glomerular endothelium dysfunction CXCL8 [79]/ recruit neutrophil infiltration and initiate inflammation CXCL9 [80,81,82,83]/ correlated with renal function CXCL10 [70,82,83]/ inhibit renal fibrosis and inflammation/ correlated with renal function (multifaceted) CXCL11 [80,82]/ correlate with baseline renal function CXCL12 [17,19,84,85]/ related to DPP-4 inhibitor associated renal protection/ recruit macrophages (multifaceted) CXCL16 [86,87,88,89]/ correlated with renal function and fibrosis	CXCR1 [78,79]/ recruit neutrophil infiltration and induce inflammation CXCR2 [78,79]/ recruit neutrophil infiltration and induce inflammation CXCR3 [81,82,83,107]/ prevent fibrosis CXCR4 [108]/ up-regulated in DN and correlated with survival signaling in tubular epithelial cell (multifaceted)
**CX3C**	CX3CL1 [90,91,92,93,112,113]/ reflect renal inflammation and pathological changes	CX3CR1 [19,93,109,110,111,112,113]/ correlated with DN progression
